# FGFR1 is critical for the anti-endothelial mesenchymal transition effect of *N*-acetyl-seryl-aspartyl-lysyl-proline via induction of the MAP4K4 pathway

**DOI:** 10.1038/cddis.2017.353

**Published:** 2017-08-03

**Authors:** Jinpeng Li, Sen Shi, Swayam Prakash Srivastava, Munehiro Kitada, Takako Nagai, Kyoko Nitta, Miyuki Kohno, Keizo Kanasaki, Daisuke Koya

**Affiliations:** 1 Department of Diabetology and Endocrinology, Kanazawa Medical University, Ishikawa, Japan; 2 Department of Pediatric Surgery, Kanazawa Medical University, Ishikawa, Japan; 3 Division of Anticipatory Molecular Food Science and Technology, Medical Research Institute, Kanazawa Medical University, Ishikawa, Japan

## Abstract

Endothelial-to-mesenchymal transition (EndMT) has been shown to contribute to organ fibrogenesis, and we have reported that the anti-EndMT effect of *N*-acetyl-seryl-aspartyl-lysyl-proline (AcSDKP) is associated with restoring expression of diabetes-suppressed fibroblast growth factor receptor (FGFR), the key anti-EndMT molecule. FGFR1 is the key inhibitor of EndMT via the suppression of the transforming growth factor *β* (TGF*β*) signaling pathway, and mitogen-activated protein kinase kinase kinase kinase 4 (MAP4K4) inhibits integrin *β*1, a key factor in activating TGF*β* signaling and EndMT. Here, we showed that the close proximity between AcSDKP and FGFR1 was essential for the suppression of TGF*β*/smad signaling and EndMT associated with MAP4K4 phosphorylation (P-MAP4K4) in endothelial cells. In cultured human dermal microvascular endothelial cells (HMVECs), the anti-EndMT and anti-TGF*β*/smad effects of AcSDKP were lost following treatment with a neutralizing FGFR1 antibody (N-FGFR1) or transfection of FRS2 siRNA. The physical interaction between FGFR1 and P-MAP4K4 in HMVECs was confirmed by proximity ligation analysis and an immunoprecipitation assay. AcSDKP induced P-MAP4K4 in HMVECs, which was significantly inhibited by treatment with either N-FGFR1 or FRS2 siRNA. Furthermore, MAP4K4 knockdown using specific siRNAs induced smad3 phosphorylation and EndMT in HMVECs, which was not suppressed by AcSDKP. Streptozotocin-induced diabetic CD-1 mice exhibited suppression of both FGFR1 and P-MAP4K4 expression levels associated with the induction of TGF*β*/smad3 signaling and EndMT in their hearts and kidneys; those were restored by AcSDKP treatment. These data demonstrate that the AcSDKP–FGFR1–MAP4K4 axis has an important role in combating EndMT-associated fibrotic disorders.

Endothelial-to-mesenchymal transition (EndMT) contributes to the appearance of fibroblasts and has a critical role in organ fibrosis.^1, 2^ In fact, EndMT is involved in various pathological processes, including kidney fibrosis,^3^ cardiac fibrosis^4^ and cancer.^5^ During EndMT, endothelial cells lose their cell-to-cell adhesion properties and acquire invasive and migratory properties.^6^ EndMT is characterized by increased expression of mesenchymal markers, such as vimentin, smooth muscle *α*-actin (*α*-SMA), smooth muscle protein 22*α* (SM22*α*) and fibroblast specific protein 1 (FSP1, also known as S100A4),^7, 8, 9, 10^ and decreased expression of endothelial cell markers, such as VE-cadherin and CD31 (also known as platelet endothelial cell adhesion molecule-1).^11, 12^ Accumulating evidence demonstrates that transforming growth factor *β* (TGF*β*)/smad signaling is a major inducer of EndMT, and proteins in the non-TGF*β* pathway, such as *β*-catenin and Notch, are also involved in EndMT induction.^13, 14^

*N*-acetyl-seryl-aspartyl-lysyl-proline (AcSDKP) is a tetrapeptide that is enzymatically cleaved by prolyl oligopeptidase (POP) from the N-terminal sequence of thymosin*β*4 (T*β*4) and hydrolyzed by angiotensin-converting enzyme.^15, 16^ Furthermore, AcSDKP has been proven to have an antifibrotic effect in various organ fibrosis models.^17, 18, 19, 20^ Long-term treatment with AcSDKP suppresses renal fibrosis by inhibiting the TGF*β*/smad signaling pathway.^21^ In the kidneys of diabetic mice, the decreased fibroblast growth factor receptor (FGFR) level was restored by AcSDKP treatment,^22^ and fibroblast growth factors (FGFs) have a key role in the normal state of vascularity.^23, 24^ FGF2/FGFR1 signaling is associated with the induction of the miRNA let-7, ameliorating the vascular disease state through the suppression of TGF*β*/TGF*β*R1-induced EndMT.^25^ Furthermore, FGFR1 is a key inhibitor of TGF*β*/smad signaling in endothelial cells.^26^ However, the molecular interplay between AcSDKP and FGFR1 signaling in endothelial cells is still unclear.

Mitogen-activated protein kinase kinase kinase kinase 4 (MAP4K4) belongs to the Ste20 family of kinases, which have been shown to affect various biological processes, such as embryonic development and inflammation. MAP4K4 is associated with multiple molecular pathways, including integrin signaling and the mammalian target of rapamycin (mTOR).^27^ In endothelial cells, MAP4K4 inhibits integrin *β*1 activity by inducing moesin.^28^ Furthermore, integrin *β*1 induces the epithelial–mesenchymal transition (EMT) and organ fibrosis via the activation of TGF*β*/smad signaling.^29, 30, 31^ We have shown that the interaction between dipeptidyl peptidase 4 (DPP-4) and integrin *β*1 induces EndMT by activating TGF*β*/smad signaling.^32^ TGF*β*/smad signaling is also inhibited by Misshapen (msn) kinases (the mammalian orthologs of MAP4K4).^33^

Here, we hypothesize that the AcSDKP–FGFR1–MAP4K4 axis has a key role in the suppression of TGF*β*/smad signaling and EndMT in endothelial cells.

## Results

### The proximity of AcSDKP and FGFR1 is essential for AcSDKP-mediated inhibition of TGF*β*/smad signaling pathway in endothelial cells

**Figure 1:**
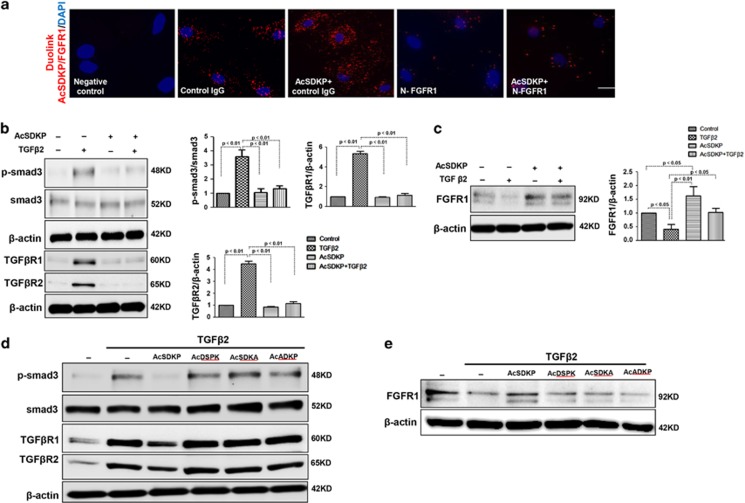
Proximity between AcSDKP and FGFR1 inhibits the TGF*β*/smad signaling pathway in HMVECs. (**a**) HMVECs were treated with N-FGFR1 (1.5  *μ*g/ml) for 48 h with or without preincubation with AcSDKP (100 nM) for 2 h, and the proximity between AcSDKP and FGFR1 was analyzed by the Duolink *In Situ* Assay. For each slide, images at a × 400 original magnification were obtained from six different areas. (**b** and **c**) HMVECs were treated with TGF*β*2 (5 ng/ml) for 15 min or 48 h with or without preincubation with AcSDKP for 2 h, and the p-smad3, TGF*β*R1, TGF*β*R2 and FGFR1 levels were analyzed by western blot. Densitometric analysis of the p-smad3/smad3, TGF*β*R1/*β*-actin, TGF*β*R2/*β*-actin and FGFR1/*β*-actin levels from each group (*n*=6) were analyzed. (**d** and **e**) HMVECs were incubated with TGF*β*2 for 15 min or 48 h with or without preincubation with AcSDKP or its mutants (AcDSPK, AcSDKA, AcADKP) (100 nM) for 2 h. The p-smad3/smad3, TGF*β*R1/*β*-actin, TGF*β*R2/*β*-actin and FGFR1/*β*-actin protein levels were analyzed by western blot

A duolink *in situ* proximity ligation assay (PLA) was performed to analyze the proximity between AcSDKP and FGFR1 in cultured human dermal microvascular endothelial cell (HMVEC) (Figure 1a). AcSDKP and FGFR1 displayed close proximity in normal cultured HMVECs (Figure 1a), suggesting that endogenous AcSDKP interacts with FGFR1. Exogenous AcSDKP incubation in endothelial cells further increased the close proximity between AcSDKP and FGFR1 (Figure 1a). In addition, in the presence of a neutralizing FGFR1 antibody (N-FGFR1), which suppresses FGFR1 activity in endothelial cells, the close proximity between AcSDKP and FGFR1 was markedly diminished in HMVECs, even with AcSDKP incubation (Figure 1a). We previously reported that the anti-TGF*β*1/smad and anti-kidney fibrosis effects of AcSDKP were associated with the restoration of FGFR phosphorylation (P-FGFR) and FGFR levels.^22, 34^ We also confirmed that AcSDKP significantly inhibited TGF*β*2-induced smad3 phosphorylation (p-smad3), p-smad2, TGF*β*R1 and TGF*β*R2 levels, and restored TGF*β*2-suppressed FGFR1 in endothelial cells (Figures 1b and c and Supplementary Figure S1a). Interferon-*γ* (IFN-*γ*) has been confirmed to inhibit FGFR1 protein levels,^25^ and treatment of HMVECs with AcSDKP was associated with increased FGFR1/P-FGFR1 levels and decreased expression of IFN-*γ* (Supplementary Figure S1b). Furthermore, T*β*4 restored TGF*β*2-suppressed FGFR1 levels, and a POP inhibitor (KYP-2047) abolished the effect of T*β*4 (Supplementary Figure S2a). The bone morphogenetic protein (BMP) pathway has been associated with EndMT induction.^35^ We investigated whether AcSDKP inhibits EndMT by suppressing the BMP pathway, and AcSDKP did not inhibit the BMP-2/BMP-4-induced expression of p-smad1/5/8 in endothelial cells (Supplementary Figure S2b). To further elucidate the significance of AcSDKP on FGFR1 levels and its functional relevance, three AcSDKP mutant peptides, AcSDKA, AcDSPK and AcADKP, were constructed. Western blot analysis revealed that the AcSDKP mutants had no effect on the levels of TGF*β*2-induced p-smad3, TGF*β*R1 and TGF*β*R2 and suppressed FGFR1 in endothelial cells (Figures 1d and e).

### AcSDKP suppresses TGF*β*/smad signaling and EndMT through the FGFR1 pathway

**Figure 2:**
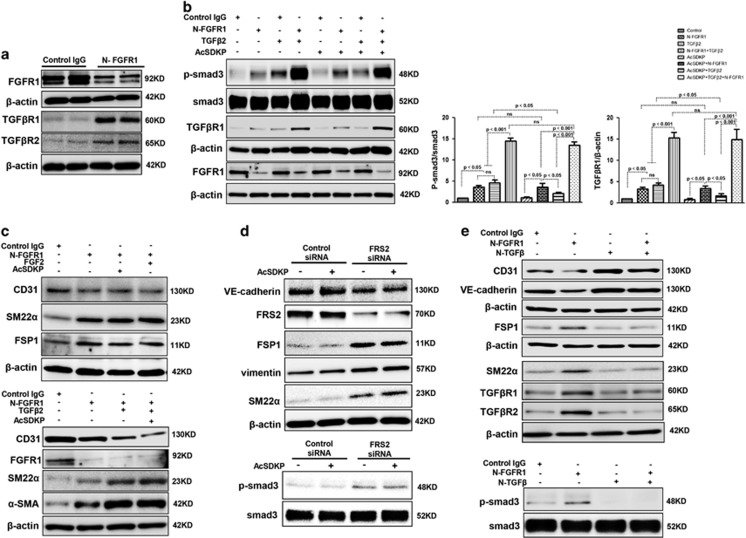
AcSDKP suppresses TGF*β*/smad signaling and EndMT through the FGFR1/FRS2 pathway. (**a**) HMVECs were treated with N-FGFR1 for 48 h, and the FGFR1, TGF*β*R1 and TGF*β*R2 protein levels were analyzed by western blot. (**b**) HMVECs were treated with TGF*β*2 in the presence or absence of N-FGFR1 for 15 min with or without AcSDKP preincubation. The p-smad3 and TGF*β*R1 protein levels were analyzed by western blot. Densitometric analysis of the p-smad3/smad3 and TGF*β*R1/*β*-actin levels (*n*=3) in each group was performed. (**c**) HMVECs were incubated with either N-FGFR1 in the presence or absence of TGF*β*2 for 48 h with or without preincubation with AcSDKP for 2 h or with N-FGFR1 in the presence or absence of TGF*β*2 for 48 h with or without 24 h of incubation with FGF2 (50 ng/ml). The CD31, SM22*α*, FSP1 and *α*-SMA protein levels were analyzed by western blot. (**d**) HMVECs were transfected with FRS2 siRNA (100 nM) for 48 h with or without AcSDKP preincubation. The VE-cadherin, FSP1, vimentin, SM22*α* and p-smad3 levels were analyzed by western blot. (**e**) HMVECs were treated with N-FGFR1 for 48 h or 15 min in the presence or absence of N-TGF*β* (1, 2, 3) (1.0  *μ*g/ml). The CD31, VE-cadherin, SM22*α*, FSP1, TGF*β*R1, TGF*β*R2 and p-smad3 levels were analyzed by western blot

N-FGFR1 suppressed FGFR1 and induced both TGF*β*R1 and TGF*β*R2 in cultured endothelial cells (Figure 2a). Treating the cultured cells with N-FGFR1 increased the p-smad3 and TGF*β*R1 protein levels, especially when N-FGFR1 and TGF*β*2 were incubated together (Figure 2b). However, in the presence of N-FGFR1, increased p-smad3 and TGF*β*R1 levels were not suppressed by AcSDKP in endothelial cells (Figure 2b), suggesting that AcSDKP-suppressed TGF*β*/smad signaling is dependent on the FGFR1 pathway. Nevertheless, AcSDKP did not bind TGF*β*R1 or TGF*β*R2 in endothelial cells (Supplementary Figure S3), likely indicating that there is no distinct interaction between AcSDKP and TGF*β*Rs. FGF2/FGFR1 has been shown to inhibit EndMT induction.^25, 26^ N-FGFR1 treatment in endothelial cells induced EndMT, as revealed by the decreased CD31 levels and the increased SM22*α*, FSP1 and *α*-SMA, and was associated with TGF*β*R1 and TGF*β*R2 levels, especially when N-FGFR1 and TGF*β*2 were applied together (Figure 2c and Supplementary Figures S4a and b). In addition, the N-FGFR1-induced EndMT was not reversed by AcSDKP or FGF2 treatment (Figure 2c and Supplementary Figures S4a and b). Knocking down fibroblast growth factor substrate 2 (FRS2), a key adaptor of FGFR1,^36^ using a specific siRNA also induced p-smad3 expression and EndMT (Figure 2d). However, AcSDKP did not suppress FRS2 siRNA-induced p-smad3 and EndMT in endothelial cells (Figure 2d). In addition, a neutralizing TGF*β* (1-2-3) antibody (N-TGF*β*) completely abolished the N-FGFR1-induced TGF*β*/smad signaling and EndMT (Figure 2e). These findings suggest that the anti-TGF*β*/smad and anti-EndMT effects of AcSDKP are dependent on the FGFR1/FRS2 signaling pathway.

### FGF2/FGFR1 regulates MAP4K4 signaling in endothelial cells

**Figure 3:**
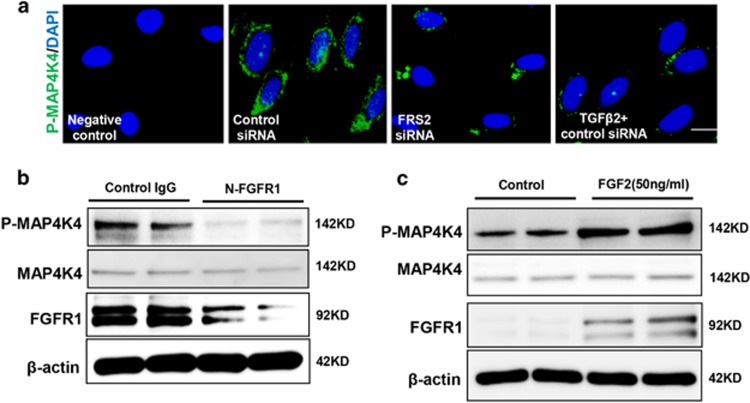
FGF2/FGFR1 mediates MAP4K4 signaling in endothelial cells. (**a**) Immunofluorescence microscopy analysis of P-MAP4K4 expression following FRS2 siRNA or TGF*β*2 treatment. For each slide, images of six different fields of view at × 400 magnification were evaluated. The scale bar is 60 *μ*m in each panel. (**b** and **c**) HMVECs were treated with N-FGFR1 for 48 h or FGF2 for 24 h. The P-MAP4K4 levels were analyzed by western blot

**Figure 4:**
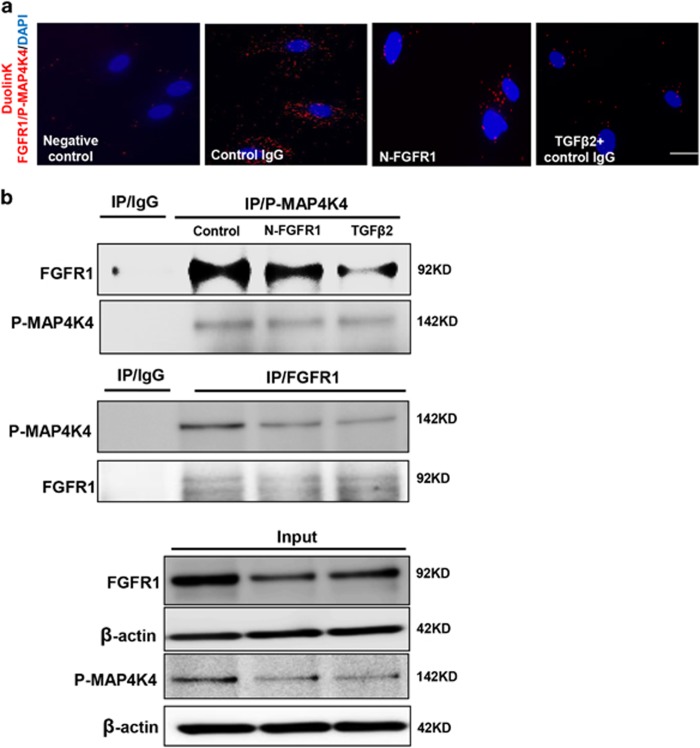
The proximity between FGFR1 and P-MAP4K4 decreases in FGFR1-deficient cells. (**a**) HMVECs were treated with N-FGFR1 or TGF*β*2 for 48 h. The proximity between FGFR1 and P-MAP4K4 was analyzed using the Duolink *In Situ* Assay. For each slide, images at × 400 original magnification were obtained from six different areas. (**b**) immunoprecipitation analysis with either a P-MAP4K4 or a FGFR1 antibody was performed and analyzed by western blot. Then, the FGFR1 and P-MAP4K4 levels with N-FGFR1 or TGF*β*2 treatment were analyzed by western blot in endothelial cells

We next investigated the mechanism responsible for decreasing the expression of phosphorylated MAP4K4 (P-MAP4K4) after the FGFR1 pathway was shut down. Immunofluorescence staining revealed that the siRNA-mediated knockdown of FRS2 in endothelial cells significantly reduced the expression of P-MAP4K4 compared with that of the control group (Figure 3a), and TGF*β*2-treated cells also showed a similar result (Figure 3a). Western blot analysis was performed to confirm these findings; the expression of P-MAP4K4 in endothelial cells was suppressed by N-FGFR1 and activated by FGF2 (Figures 3b and c). The close proximity between FGFR1 and P-MAP4K4 was observed in normal cultured endothelial cells (Figure 4a), and this close proximity was significantly decreased in the N-FGFR1-treated cells (Figure 4a); TGF*β*2 treatment also produced comparable results (Figure 4a). Furthermore, PF-06260933, a potent MAP4K4 inhibitor, significantly suppressed the proximity between FGFR1 and MAP4K4 in endothelial cells (Supplementary Figure S5), suggesting that MAP4K4 phosphorylation is essential for the proximity between FGFR1 and MAP4K4. Immunoprecipitation assays revealed a physical interaction between FGFR1 and P-MAP4K4, and N-FGFR1 decreased this interaction in endothelial cells (Figure 4b).

### MAP4K4 signaling is activated by AcSDKP in a FGFR1/FRS2-dependent manner

**Figure 5:**
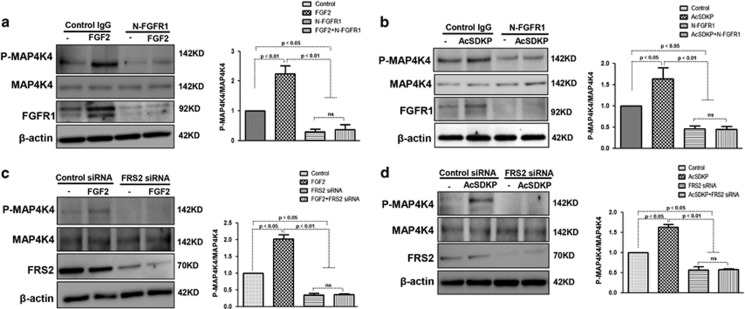
MAP4K4 signaling is mediated by AcSDKP in a FGFR1/FRS2-dependent manner. (**a** and **b**) HMVECs were treated with N-FGFR1 for 48 h in the presence or absence of FGF2 or AcSDKP. P-MAP4K4 levels were analyzed by western blot. Densitometric analysis of P-MAP4K4 levels normalized to MAP4K4. For each group, *n*=3 were analyzed. (**c** and **d**) HMVECs were transfected with FRS2 siRNA for 48 h with or without FGF2 or AcSDKP treatment. P-MAP4K4 levels were analyzed by western blot. Densitometric analysis of P-MAP4K4 levels, normalized to MAP4K4. A total of *n*=3 from each group were analyzed

We next examined whether AcSDKP-activated MAP4K4 signaling in endothelial cells via the FGFR1/FRS2 pathway. Endothelial cells were treated with either N-FGFR1 or FRS2 siRNA in the presence or absence of FGF2 or AcSDKP. In the presence of N-FGFR1, the P-MAP4K4 levels were significantly decreased in endothelial cells and could not be restored by FGF2 (Figure 5a). Similarly, AcSDKP also failed to restore the N-FGFR1-suppressed P-MAP4K4 levels (Figure 5b). Furthermore, in FRS2 siRNA-treated endothelial cells, neither AcSDKP nor FGF2 restored FRS2 siRNA-suppressed P-MAP4K4 levels (Figures 5c and d). These findings suggest that FGFR1 is a key upstream molecule of MAP4K4 signaling in endothelial cells.

### MAP4K4 is a key inhibitor of TGF*β*/smad signaling and EndMT via suppression of integrin *β*1 signaling

**Figure 6:**
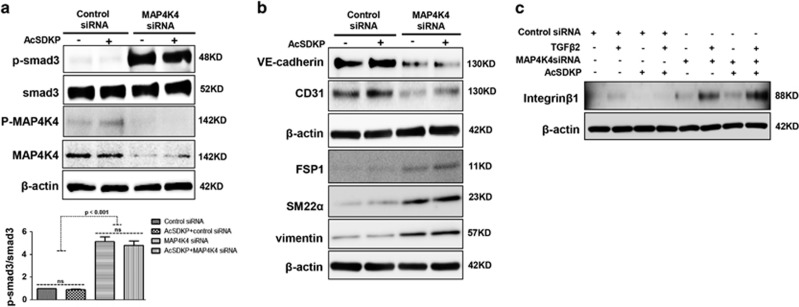
MAP4K4 deficiency induces TGF*β*/smad signaling and EndMT via activation of integrin *β*1. (**a**) HMVECs were transfected with MAP4K4 siRNA (100 nM) for 48 h. Next, the cells were treated with or without AcSDKP for 2 h. The p-smad3/smad3 pathway was analyzed by western blot. Densitometric analysis of the p-smad3/smad3 levels was performed, with *n*=3 for each group. (**b**) HMVECs were treated with MAP4K4 siRNA for 48 h with or without AcSDKP treatment. The VE-cadherin, CD31, FSP1, SM22*α* and vimentin protein levels were analyzed by western blot. (**c**) HMVECs were transfected with MAP4K4 siRNA for 48 h in the presence or absence of TGF*β*2 with or without AcSDKP. The integrin *β*1 level was analyzed by western blot

In endothelial cells, the increased p-smad3 level induced by MAP4K4 siRNA was not suppressed by AcSDKP treatment (Figure 6a). In addition, MAP4K4 siRNA treatment led to decreased expression of the endothelial cell markers VE-cadherin and CD31 and increased expression of the mesenchymal markers FSP1, SM22*α* and vimentin (Figure 6b), suggesting that MAP4K4 deficiency induces EndMT. However, AcSDKP did not inhibit the MAP4K4 siRNA-induced EndMT (Figure 6b). MAP4K4 has been shown to inhibit the expression of integrin *β*1 in endothelial cells,^28^ and we have previously reported that the interaction between DPP-4 and integrin *β*1 can induce EndMT.^32^ In this study, both TGF*β*2 and MAP4K4 siRNA increased the expression of integrin *β*1 in endothelial cells, especially when TGF*β*2 and MAP4K4 siRNA were applied together (Figure 6c). However, in the cells in which MAP4K4 was knocked down, AcSDKP failed to restore the increased levels of integrin *β*1 (Figure 6c).

### AcSDKP inhibits diabetes-induced EndMT and restores diabetes-suppressed FGFR1 and P-MAP4K4 expression in mice

**Figure 7:**
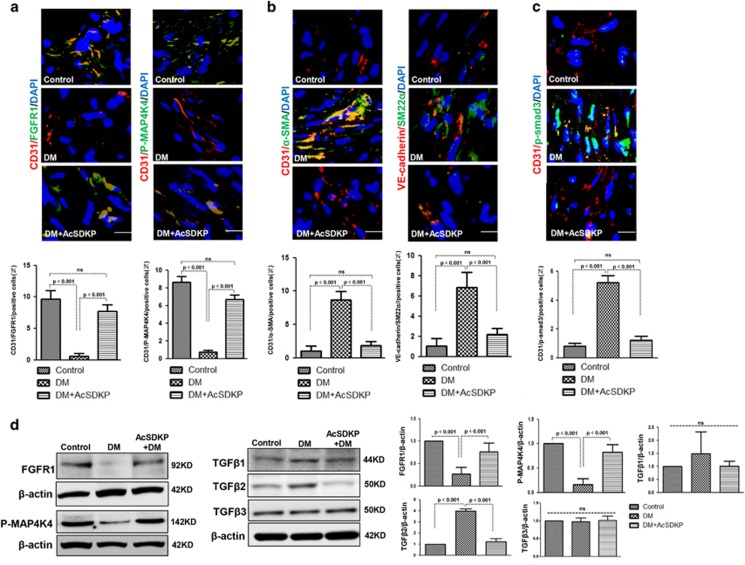
AcSDKP inhibits TGF*β*/smad signaling and EndMT and restores the FGFR1 and P-MAP4K4 levels in diabetic hearts. (**a**) Immunofluorescence microscopy analysis of CD31/FGFR1 and CD31/P-MAP4K4 in the heart tissues from each group of mice. The scale bar is 60 *μ*m in each panel. The CD31 and FGFR1 double-labeled cells and the CD31 and P-MAP4K4 double-labeled cells in each visual field were assessed by fluorescence microscopy and quantified. For each section, images from six different fields of view at × 400 magnification were evaluated. (**b** and **c**) Immunofluorescence microscopy analysis of CD31/*α*-SMA, VE-cadherin /SM22*α* and CD31/p-smad3 expression levels in the heart tissues from each group of mice. The scale bar is 60 *μ*m in each panel. The CD31 and *α*-SMA double-labeled cells, the VE-cadherin and SM22*α* double-labeled cells and the CD31 and p-smad3 double-labeled cells in each visual field were analyzed by fluorescence microscopy and quantified. For each section, images from six different fields of view at × 400 magnification were evaluated. Four mice from each group were analyzed. (**d**) Western blot analysis of the FGFR1, P-MAP4K4, TGF*β*1, TGF*β*2 and TGF*β*3 levels in cardiac tissues. A representative blot from four independent experiments was shown. The densitometric analysis of western blot data was presented (*n*=4). The diabetic mice are abbreviated as DM in the figure

AcSDKP restored the high glucose (HG)-induced EndMT in cultured endothelial cells (Supplementary Figure S7). Therefore, to investigate the role of the AcSDKP–FGFR1–MAP4K4 signaling pathway in EndMT induction *in vivo*, we developed AcSDKP-treated streptozotocin (STZ)-induced diabetic mice. FGFR1 and P-MAP4K4 expression in their cardiac tissue endothelial cells was analyzed by immunofluorescence, and FGFR1 and P-MAP4K4 expression was markedly suppressed in the diabetic hearts compared with the control group (Figure 7a). In addition, AcSDKP treatment restored the diabetes-suppressed endothelial FGFR1 and P-MAP4K4 expression (Figure 7a). We next analyzed endothelial cells in hearts undergoing EndMT, which were recognized by double labeling with CD31/*α*-SMA or VE-cadherin/SM22*α* antibodies. The diabetic hearts exhibited more endothelial cells undergoing EndMT compared with the control hearts (Figure 7b), and AcSDKP treatment restored the diabetes-induced EndMT (Figure 7b). When compared with the control mice, the diabetic mice exhibited increased endothelial p-smad3 levels in the hearts, which was inhibited by AcSDKP (Figure 7c). In the hearts, AcSDKP reversed the diabetes-suppressed FGFR1 and P-MAP4K4 levels and the diabetes-induced TGF*β*2 levels (Figure 7d). However, the TGF*β*1 and TGF*β*3 protein levels were not significantly altered (Figure 7d). In kidneys, AcSDKP also restored the diabetes-suppressed FGFR1 and P-MAP4K4 levels and the diabetes-induced TGF*β*1 and TGF*β*2 expression (Supplementary Figure S6). The TGF*β*3 protein level was not significantly altered (Supplementary Figure S6).

## Discussion

In our study, we found that AcSDKP inhibited TGF*β*/smad signaling and EndMT in endothelial cells via the FGFR1/MAP4K4 pathway *in vitro* and *in vivo*. *In vitro*, TGF*β*/smad signaling and the associated EndMT were induced by FGFR1 deficiency in a TGF*β*2-dependent manner. AcSDKP-activated MAP4K4 signaling through the FGFR1/FRS2 pathway. AcSDKP could not suppress the TGF*β*/smad signaling and EndMT induced by MAP4K4 deficiency. *In vivo*, we also confirmed that AcSDKP restored the expression of FGFR1 and P-MAP4K4 and was associated with TGF*β*/smad3 expression and EndMT in diabetic hearts and kidneys.

**Figure 8:**
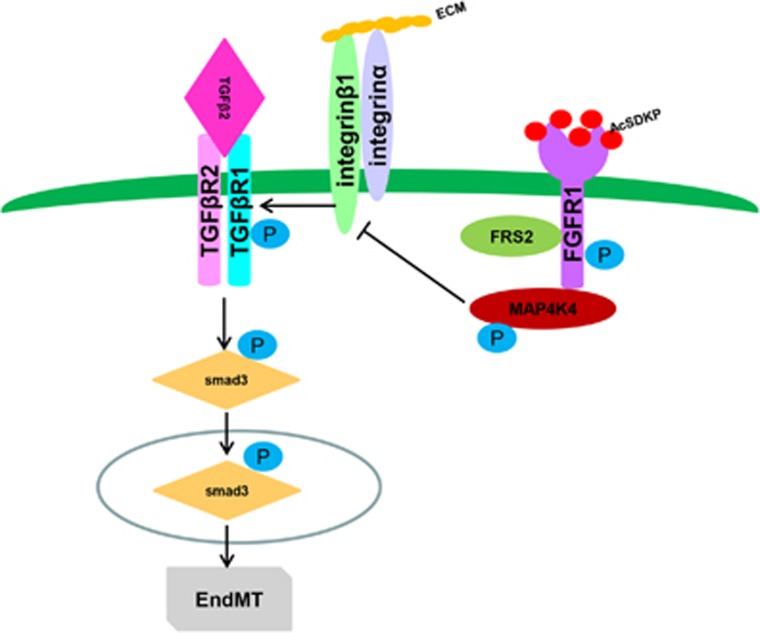
Schematic of the AcSDKP/FGFR1/MAP4K4 pathway suppression of TGF*β*/smad signaling and EndMT. In endothelial cells, the close proximity between AcSDKP and FGFR1 increased FGFR1 and induced its phosphorylation levels. Interacting with co-factor FRS2, FGFR1 recruited MAP4K4 and induced its phosphorylation. Subsequently, p-MAP4K4 suppressed integrin*β*1 (integrin*β*1 should be localized on the cell surface interacted with some of *α* integrins). Integrin*β*1 was a potent activator of TGF-*β* signaling and also EndMT. Therefore, AcSDKP could inhibit EndMT through FGFR1-MAP4K4-dependent manner

AcSDKP exhibits anti-EndMT and antifibrotic effects in several organ fibrosis models,^22, 37^ and EndMT has shown to be induced by TGF*β*2 in both a smad-dependent and a smad-independent manner.^11, 13, 38^ AcSDKP has also been shown to inhibit TGF*β*1-induced p-smad2/3.^34^ However, the detailed molecular mechanisms by which AcSDKP inhibits TGF*β*/smad signaling and EndMT in endothelial cells are not entirely clear. A recent study confirmed that FGF/FGFR suppressed TGF*β*-induced EndMT via the induction of miR-let-7s.^25^ Our previous study also demonstrated that AcSDKP restored diabetes-suppressed FGFR and FGFR phosphorylation levels.^22^ FGFR1 has been confirmed as a key inhibitor of TGF*β*-induced EndMT.^26^ Here, we established the FGFR1 signaling-dependent inhibitory effects of AcSDKP on TGF*β*/smad signaling and EndMT in HMVECs (Figure 8). AcSDKP exhibited anti-TGF*β*/smad3 and anti-EndMT effects and restored the TGF*β*2-suppressed FGFR1 levels. AcSDKP also suppressed IFN-*γ*, the potent molecule that inhibited FGFR1.The anti-TGF*β*/smad and anti-EndMT effects of AcSDKP were lost when FGFR1 signaling was disrupted.

FGFR1 has a key role in the regulation of cell migration,^39, 40, 41^ and the interaction between FGFR1 and integrin *β*1 is essential for endothelial cell migration.^42^ In addition, MAP4K4 promotes endothelial cell migration via inactivation of integrin *β*1 signaling.^28^ Here, we confirmed that FGFR1 was essential for regulation of the MAP4K4 pathway in endothelial cells, as suppression of FGFR1 clearly decreased P-MAP4K4 levels. Conversely, FGF2-induced FGFR1 activation was associated with the induction of P-MAP4K4 (Figure 8).

Kaneko *et al.*^33^ reported that misshapen, the mammalian ortholog of MAP4K4, was a direct suppressor of the TGF*β*/smad pathway. Recent studies showed that MAP4K4 deficiency in T cells led to insulin resistance and that MAP4K4 expression was significantly suppressed in type 2 diabetic mice.^43, 44^ Despite these important biological roles, little information is known regarding the regulation of MAP4K4 in TGF*β*/smad signaling and EndMT induction. Our data demonstrated that AcSDKP-activated P-MAP4K4 expression was dependent on the FGFR1 signaling pathway. In addition, the suppression of MAP4K4 in endothelial cells significantly induced TGF*β*/smad signaling and EndMT, even in the presence of AcSDKP (Figure 8). *In vivo*, we demonstrated that AcSDKP reversed the diabetes-suppressed FGFR1 and P-MAP4K4 levels associated with the induction of TGF*β*/smad signaling and EndMT in endothelial cells in hearts and kidneys.

MAP4K4 has been shown to suppress integrin *β*1 signaling in endothelial cells^28^ and integrin *β*1 is associated with TGF*β* signaling and EndMT induction in endothelial cells.^32, 45^ In our study, suppression of MAP4K4 increased the integrin *β*1 levels in endothelial cells, and AcSDKP had no effect on its levels in MAP4K4-deficient cells (Figure 8). These data confirmed that MAP4K4 is a key downstream molecule for the anti-EndMT effect of AcSDKP.

Our results confirmed the functional interactions among AcSDKP, FGFR1 and MAP4K4 in endothelial cells. The close proximity between AcSDKP and FGFR1 inhibited the EndMT associated with TGF*β*/smad signaling by activating the MAP4K4 pathway. In addition, AcSDKP restored both diabetes-suppressed FGFR1 and P-MAP4K4 levels and induced TGF*β*/smad signaling and EndMT in heart and kidney. These findings reveal an AcSDKP–FGFR1–MAP4K4 signaling axis, offering new insights into endothelial cell biology and a potential target for future studies of EndMT-associated organ fibrosis.

## Materials and methods

### Reagents

The AcSDKP peptide was a gift from Asabio Bio Technology (Osaka, Japan). The AcSDKA (100 nmol, SQ14640), AcDSPK (100 nmol, SQ14641) and AcADKP (100 nmol, SQ14639) peptides were purchased from Scrum Inc. (Tokyo, Japan). The following antibodies were purchased from Abcam (Cambridge, UK): mouse monoclonal anti-FGFR1 (1 :  500, ab823), rabbit polyclonal anti-integrin *β*1(1 : 500, ab115146), mouse monoclonal anti-vimentin (1 : 2000, ab8978), rabbit polyclonal anti-*α*-SMA (1 : 1000, ab5694), rabbit monoclonal anti-IFN-*γ* (1 : 1000, ab133566), rabbit polyclonal anti-TGF*β*RII (1 : 1000, ab61213) and rabbit polyclonal anti-TGF*β*3 (1 : 500, ab 15537). The human BMP-4 peptide (1 *μ*g/ml, ab40140) and the human T*β*4 peptide (1 *μ*g/ml, ab42293) were also purchased from Abcam. The mouse monoclonal anti-human CD31 (1 : 1000, AF3628), human neutralizing FGFR1 (1 : 500, MAB765) and neutralizing TGF*β* (1-2-3) (1 : 500, MAB1835) antibodies were purchased from R&D Systems (Minneapolis, MN, USA). A rabbit polyclonal anti-phospho-smad3 (s423 and s425) (1 : 1000, 600-401-919) antibody was purchased from Rockland Immunochemicals (Gilbertsville, PA, USA). The mouse monoclonal anti-*β*-actin (1:10 000, A2228), rabbit anti-TGF*β*1 (1:500, SAB4502954) and rabbit anti-TGF*β*RI (1:500, SAB4502958) antibodies, as well as FGF2 human recombinant (50 *μ*g/ml, SRP4037), KYP-2047 (1 *μ*g/ml, SML0208) and PF-06260933 (1 *μ*mol, PZ-0272) were obtained from Sigma (St. Louis, MO, USA). The rabbit polyclonal anti-phospho-HGK/MAP4K4 PSer801 antibody (1:500, PA5-12874) and the rabbit anti-SMAD2 (1 : 500, #51-1300) antibody were purchased from Thermo Fisher Scientific (Waltham, MA, USA). The rabbit polyclonal anti-HGK/MAP4K4 (1 : 500, NBP1-58853), rabbit polyclonal anti-SM22*α* (1:1000, NBP1-33003) and rabbit monoclonal anti-VE-cadherin (1:1000, NBP1-43347) antibodies, along with the recombinant human BMP-2 protein (1 *μ*g/ml, NBP1-98923) were obtained from Novus Biologicals (Littleton, CO, USA). The rabbit polyclonal anti-S100A4 (also known as FSP1) (1:200, PRB-497P), rabbit polyclonal anti-FRS2 (1:1000, sc-8318) and goat polyclonal anti-p-smad1/5/8 (1 : 1000, sc-12353) antibodies were purchased from Santa Cruz Biotechnology (Dallas, TX, USA). The rabbit polyclonal anti-AcSDKP (1 : 1000, A03881) antibody was purchased from Biocompare (South, San Francisco, CA, USA). The rabbit polyclonal anti-smad3 (1:1000, #9513), rabbit polyclonal anti-p-smad2 (1:400, #3108S) and rabbit anti-phospho-FGFR1 (1:500, #3471) antibodies were obtained from Cell Signaling Technology (Danvers, MA, USA). The TGF*β*2 antibody (1:1000, GTX15539) was purchased from Gene Tex (Alton Pkwy, Irvine, CA, USA).

### Cell culture and treatment

The HMVECs were cultured in EBM-2 medium supplemented with EGM-2 (5.5 mmol/l glucose, fetal bovine serum, hydrocortisone, hFGF-*β*, VEGF, R-IGF-1, ascorbic acid, hEGF, GA-1000 and heparin) (Lonza, Alpharetta, GA, USA). When the cells reached 70–80% confluence, TGF*β*2 (5 ng/ml), N-FGFR1 (1.5 *μ*g/ml) and TGF*β*2 with either the N-FGFR1 or the neutralizing TGF*β* (1, 2, 3) antibody (1.0 *μ*g/ml) was added to the experimental medium (a mixture of HuMedia-MVG in serum-free RPMI 1640 medium at a 1 : 3 ratio) with or without preincubation with 100 nM AcSDKP for 2 h.

### Transfection experiments

HMVECs were transfected with siRNA (100 nmol/l) targeting FRS2 (Invitrogen, Carlsbad, CA, USA) or MAP4K4 (Dharmacon, Lafayette, CO, USA) (FRS2: 5′-GCGGAAAAACCGCAAGUTT-3′ and MAP4K4: 5′-GACCAACUCUGGCUUGUUA-3′). The transfection reagent lipofectamine 2000 (Invitrogen) was used according to the manufacturer’s instructions. Cells were incubated with lipofectamine 2000 and target RNA in serum-free medium for 6 h. After this, the medium was replaced by experimental medium (a mixture of serum-free Humedia-EB2 in serum-free RPMI at a 1:3 ratio), the cells were treated as follows: for FRS2 siRNA transfection, the cells were treated with or without FGF2 (50 ng/ml) for 24 h or AcSDKP for 48 h; for the MAP4K4 siRNA transfection, the cells were treated with TGF*β*2 for 48 h in the presence or absence of AcSDKP.

### Duolink *in situ* assay

The duolink *in situ* PLA was performed according to the manufacturer’s protocol. Briefly, HMVECs were treated with N-FGFR1 (1.5 ug/ml) or MAP4K4 siRNA (100 nmol) for 48 h with or without preincubation with AcSDKP. Control IgG was added in the negative control group, and TGF*β*2 and control IgG were added in the positive control group. Next, the cells were fixed with 4% paraformaldehyde and permeabilized with 0.2% Triton X-100. After blocking, the cells were incubated overnight with rabbit anti-AcSDKP (1 : 1200) and mouse anti-FGFR1 (1 : 50) or mouse anti-FGFR1 (1 : 50) and rabbit anti-P-MAP4K4 (1 : 1200). The cells were incubated with PLA probe solution for 1 h at 37 °C before being treated with a ligase solution for 30 min at 37 °C and a polymerase amplification solution for 100 min at 37 °C. The samples were immediately mounted with Duolink *In Situ* Mounting Medium with DAPI for 20 min and analyzed by fluorescence microscopy. For each slide, the images were analyzed from six different view fields at a × 400 magnification.

### Western blot analysis

Proteins were harvested using RIPA lysis buffer (lysis buffer, PMSF, protease inhibitor cocktail and sodium orthovanadate, which was purchased from Santa Cruz Biotechnology). The protein lysates were boiled in SDS sample buffer at 100 °C for 6 min, separated on SDS-polyacrylamide gels, and then transferred onto PVDF membranes (Pall Corporation, Pensacola, FL, USA) using the semi-dry method. After blocking, the membranes were incubated overnight with primary antibodies at 4 °C, followed by the corresponding peroxidase-conjugated secondary antibody for 1 h at room temperature. The blots were developed with an enhanced chemiluminescence detection system (Pierce Biotechnology, Rockford, IL, USA) and visualized using an ImageQuant LAS 400 camera system (GE Healthcare Life Sciences, Uppsala, Sweden).

### Immunoprecipitation

HMVECs were treated with either N-FGFR1 or TGF*β*2. After 48 h, the cells were harvested using the RIPA lysis buffer system and centrifuged at 14 000 × *g* for 15 min at 4 °C. The supernatant was then transferred to a new tube, washed twice with protein A solution (Cell Signaling Technology), and incubated with primary antibody overnight at 4 °C. Protein A was added again, and incubated for 2 h at 4 °C. Next, the solution was centrifuged, and the supernatant was discarded. The pellets were washed three times with RIPA lysis buffer. After the supernatant was discarded, 2 × SDS sample buffer was added to the pellets, and the mixture was boiled for 5 min to elute the captured proteins for western blotting.

### Immunofluorescence for cell culture

The treated HMVECs were cultured on eight-well culture slides for 48 h. The cells were then fixed with 100% methanol for 10 min at −20 °C and acetone for 1 min at −20 °C. After blocking with 2% BSA/PBS for 30 min at room temperature, the cells were incubated with primary antibody for 1 h. After being washed with PBS, the slides were incubated with the corresponding secondary antibodies for 30 min. The cells were then extensively washed three times with PBS and mounted with mounting medium containing DAPI (Vector Laboratories, Burlingame, CA, USA). The images were analyzed by fluorescence microscopy (Axio Vert.A1, Carl Zeiss Microscopy GmbH, Jena, Germany).

### Immunostaining analysis of mouse tissue

Frozen heart sections were fixed with acetone for 10 min at −20 °C. The sections were blocked with 2% BSA/PBS for 30 min at room temperature and incubated with primary antibodies against CD31/FGFR1, CD31/P-MAP4K4, CD31/*α*-SMA, VE-cadherin/SM22*α* and CD31/p-smad3 for 1 h. The sections were then incubated with the corresponding secondary antibodies for 30 min at room temperature and mounted in mounting medium containing DAPI. The images were analyzed by fluorescence microscopy (Axio Vert.A1, Carl Zeiss Microscopy GmbH).

### Animal experiments

Control, STZ-treated CD-1 and AcSDKP-treated STZ mice were prepared based on our previous report.^22^ Briefly, 8-week-old CD-1 mice were injected with STZ (200 mg/kg). Sixteen weeks after the induction of diabetes, the diabetic mice were divided into two groups, a nontreatment group and an AcSDKP treatment group (500 *μ*g/kg BW/day using an osmotic mini-pump for 8 weeks). The experiments described in the methods were carried out in accordance with the animal protocols of Kanazawa Medical University (protocol numbers 2014-89, 2013-114 and 2014-101).

### Statistical analysis

The data are expressed as the mean±S.E.M. One-way ANOVA followed by the Tukey’s multiple comparison test (statistical significance was defined as *P*<0.05) was used for statistical analysis using GraphPad Prism software (Ver 5.0f, La Jolla, Canada).
